# Suicide in the press: an analysis of newspaper coverage of adolescent versus adult suicides in Italy

**DOI:** 10.1192/j.eurpsy.2024.2

**Published:** 2024-01-17

**Authors:** Chiara Davico, Luca Arletti, Giulia Silverio, Daniele Marcotulli, Federica S. Ricci, Federico Amianto, Benedetto Vitiello

**Affiliations:** 1 Section of Child and Adolescent Neuropsychiatry, Department of Public Health and Pediatric Sciences, University of Turin, Turin, Italy; 2 Department of Neuroscience, University of Turin, Turin, Italy; 3 Department of Mental Health, School of Public Health, Johns Hopkins Bloomberg School of Public Health, Baltimore, MD, USA

**Keywords:** adolescents, media, press, prevention, suicide

## Abstract

**Background:**

An association between sensationalized media reporting and subsequent increase in suicidal behavior has been documented, and adolescents are especially vulnerable to imitative influences. The aims of this study were to examine the characteristics of the articles reporting adult and adolescent (under age 18) suicides in the Italian press and to assess adherence to the World Health Organization (WHO) guidelines for responsible reporting.

**Methods:**

The print versions of the three newspapers with the widest national distribution in Italy were searched for all the articles on incident suicides printed over a 7-month period (July 2022 to February 2023). Articles were examined for adherence to the WHO guidelines.

**Results:**

Overall, 213 articles were identified, reporting on 122 individual suicide cases (88.5% adults and 11.5% adolescents). Of the articles, 78.9% were on adults and 21.1% on adolescents, with a ratio articles/suicide cases of 1.6 for adults and 3.2 for adolescents (*p* < 0.0001). Adolescent suicide articles had more words (mean 612.5 ± SD 275.6) than adult ones (462.1 ± 267.7, *p* = 0.001). Potentially harmful reporting features were present in both the adult and adolescent articles (12–82%). Few articles (0–15%) included protective features. Articles on adolescents were more adherent to the WHO guidelines for omitting specific information of suicide method and location.

**Conclusions:**

Significant differences were found in the press reporting of adolescent versus adult suicides, with adolescent suicides receiving more attention in terms of the number of articles and article length. Suicide press reporting can be improved. A close collaboration between journalists and suicide prevention experts may be beneficial.

## Introduction

Suicide is a major public health concern worldwide, with about 700,000 people dying of suicide every year [1]. Even if only a small proportion of all suicides occurs in adolescence, suicide is a leading cause of death in youth, ranking second after unintentional injuries in economically developed countries [2, 3]. In the United States, only 1% of all deaths in the population is among adolescents (12–19 years of age), but about 4% of all suicides are in this age-group [2, 4]. While the incidence of suicide shows considerable between-country variability, male suicide rate consistently exceeds by several folds the female rate [1–3]. In Italy, the estimated suicide rate is 2.58 per 100,000 subjects aged 15–19 years, with a male:female ratio of 3.13 [3]. Increasing rates of adolescent suicide and suicidal behaviors (including suicidal ideation and suicide attempts) have been documented over recent years, starting prior to the COVID-19 pandemic and then continuing afterward [5–8].

Prevention of suicide must consider the many relevant risk and protective factors and the complexity of their interaction [9]. Among the risk factors, the existence of a form of social contagion (“copycat” phenomenon) has been reported [10–12]. The role of the media in contributing to suicide contagion in the population has been studied, with the conceptualization of a potentially detrimental effect (the *Werther* effect) but also of a potential protective effect (the *Papageno* effect) from exposure to suicide coverage [13]. In this regard, the news about the suicide of celebrities has been found to be especially influential [14, 15]. People with depression and those with a history of self-harm are more vulnerable to these imitation effects, and the impact is greater on people with similar gender–age characteristics as the deceased [13–18]. The risk of imitation seems to be increased by spectacular and graphic reporting techniques, such as giving a detailed description of the suicide method [19].

Youth are especially vulnerable to imitative influences, being developmentally more sensitive to social learning and prone to modeling behavior. Adolescents have a greater tendency to identify with models perceived as similar or admirable. These processes are evident also with suicidal behavior. In fact, clustering of suicidal behavior has been found to be more common in youth than in adults [15, 20, 21]. The mechanisms behind this greater sensitivity are multifold and based on the cognitive and emotional development in adolescence. Special attention to the suicide victim may be interpreted as a sign that suicide serves a purpose. Frequent and detailed reports of suicidal acts can also normalize the behavior and thus reduce inhibition, especially in adolescence when impulse control is still immature Furthermore, explicit descriptions of suicide methods can inadvertently inform readers about the most lethal means of self-harm.

Gould and colleagues found that the suicide risk of 15- to 19-year-olds exposed to reports of suicide in the United States was 2 to 4 times higher than in the other age-groups [21]. In addition, both the extent and the modalities of the newspaper coverage were significantly associated with suicide clusters, especially when the suicide news had a prominent display, such as a front-page placement, presence of pictures, headlines containing the word suicide, details on the suicide method used, and publicizing specific names [21]. An association was found between highly publicized teen suicides and an increase in the number of mental health presentations to the pediatric emergency department in Canada [22].

There are also indications that the general public’s understanding and attitudes toward suicide and its prevention can be positively influenced by the media [23–26] and that appropriate reporting can increase help-seeking and decrease suicidality [27–29].

Given the relevance of media reporting for suicide prevention, the World Health Organization (WHO) has developed guidelines for responsible reporting of suicide by the media, with a list of “dos” and “don’ts” practices [30]. Adherence to these recommendations has been examined in a number of cultural contexts and found to be generally rather limited [31–38]. Sensationalistic ways of reporting suicide, with inclusion of specific details on the method used and the location, are still encountered, together with a lack of providing practical information on how and where to seek help. However, there are also indications that responsible media reporting can indeed have a positive impact on suicide prevention. For example, a greater adherence to the media guidelines was associated with lower suicide rates in Austria [26].

**Figure 1.**
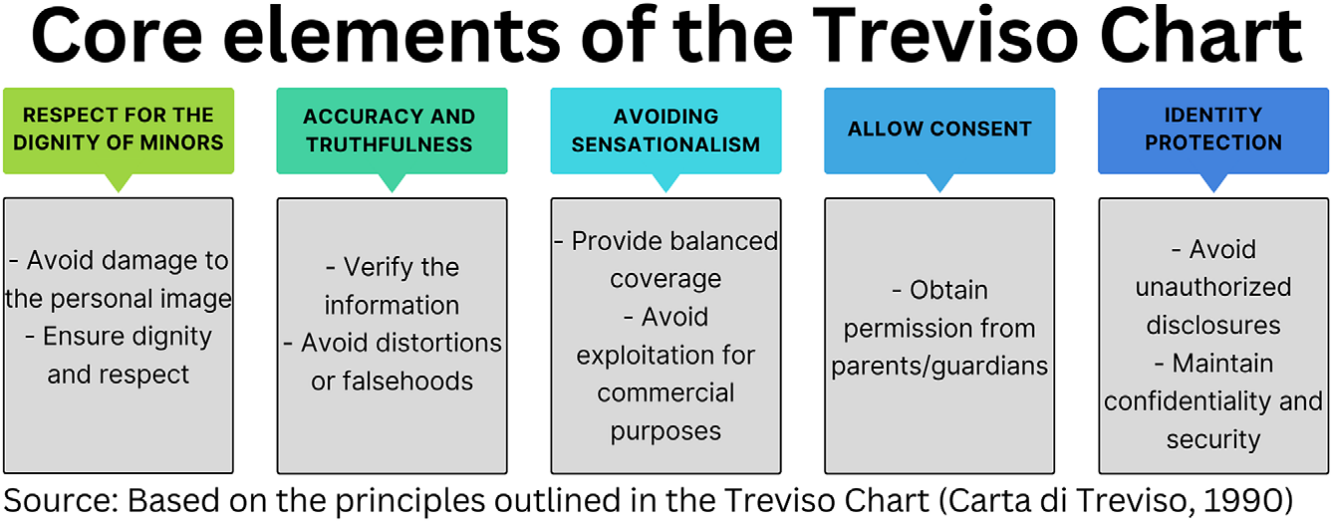
Core Elements of the Treviso Chart

In Italy, the complex relationship between media and child protection is informed by the Treviso Chart (*Carta di Treviso*), which is a document by the Italian Professional Association of Journalists and the National Federation of Italian Press (FNSI), released in 1990 and subsequently updated, as a binding self-regulation for journalists, as well as an ideal and practical guide for the whole professional category of communicators [39]. The chart represents a reference for the media reporting of news involving minors in Italy. Although not specifically focused on suicide, the chart applies to the media the principles of the United Nations Convention on the Rights of the Child [40] and requires journalists to give priority to the rights of the minors over other commercial considerations (Figure 1).

Little is known about how newspapers report adolescent suicide and if there are differences between adolescent and adult suicide reports. A better understanding of how suicide cases involving adolescents are communicated to the general public by the press may help identify areas of potential improvement and thus contribute to the efforts to prevent suicide. Italy has a relatively low rate of suicide, including adolescent suicide, as compared with most other European countries and the United States [3]. This might be explained by a number of factors, such as lower access to lethal means (particularly firearms), cultural, religious, and family structure characteristics, lower economic inequalities, and, possibly, easier access to mental health services. In addition, possible underreporting of suicide due to stigma in less developed part of the country has been considered [41].

In any case, also in Italy, concern has been raised about an increase in suicidality in recent years and especially since the COVID-19 pandemic [42].

The aim of this study was to assess the extent and modalities of press coverage of adolescent as compared with adult suicides in Italy. Specifically, the number of articles, their length, prominent placement in the newspaper, and presence of details, such as place, person, method used, and pictures, were evaluated with a descriptive approach aimed at identifying similarities and differences between adult and adolescent suicide reports. In addition, compliance to the WHO suicide reporting guidelines was evaluated.

## Methods

### Article search

The three newspapers with the highest circulation in Italy (*Il Corriere Della Sera*, *La Repubblica*, and *La Stampa*) were searched for all the printed articles reporting on instances of suicide published during the 8-month period between July 13, 2022, and February 13, 2023. According to a July 2022 analysis of the press circulation, these three newspapers accounted cumulatively for 51% of all the printed newspaper copies in Italy (from the *Accertamenti e Diffusione Stampa ADS* at https://www.adsnotizie.it/Home/Index). At the time of the search, the average daily was 222,016 copies for *Il Corriere Della Sera*, 152,690 copies for *La Repubblica*, and 133,867 copies for *La Stampa.* Both the national and local editions of these newspapers were searched. The 7-month length of observation was determined by the available funds in the research project, given that access to the newspapers required a paid subscription, but also considered to be sufficiently representative of press reporting and also consistent with the time frame used in other studies of media reporting and suicide [33, 36].

The initial search of all the articles reporting on suicide was conducted by Presstoday Ltd, an Italian press service based in Milan, Italy, under a paid service contract. The two search words were *suicidio* (suicidio) and *suicidio* (suicides). Presstoday returned daily all the news stories containing these search words. Two trained raters (L.A. and G.S.) reviewed together the articles and selected those reporting on specific suicide events, including suicide, attempted suicide, suspected suicide, and murder–suicide. Excluded were articles covering suicide as a general social, cultural, or health issue, assisted suicide, occurring in the context of terrorism, or when the word *suicide* was used as a metaphor (e.g., *political suicide*).

### Assessment process

Information about name, sex, and age of the suicide victim was extracted from each article. The suicidal event associated to each article was identified, and the number of articles reporting on each suicidal event was computed. The placement of the article within the newspaper (i.e., national or local edition, page number) and features such as presence of the word *suicide* in the title, details on the suicide modality, place, and social and personal context were recorded.

Potentially harmful or protective features of each article were examined based on the WHO and International Association for Suicide Prevention (IASP) guidelines for responsible media reporting [30]. A description of the variables collected for each article, comprising potentially harmful and protective factors, is provided in Supplementary Table 1.

Two researchers (G.S. and L.A.) independently evaluated 10% of the articles, documenting a high level of agreement between the two raters (Cohen’s κ exceeded 0.82 across all the quality ratings). Afterward, one researcher (G.S.) made the qualitative ratings, extracted the descriptive information from each article, and input the information into an Excel database created for this purpose.

### Statistical analysis

Descriptive statistics were applied to the data. Group differences were tested with chi-square test for categorical variables and with *t*-test for independent samples for continuous variables. Statistical significance was set at a two-tailed *p* < 0.05. Being an exploratory rather than a hypothesis-testing study, no control for multiple tests was applied. Inter-rater reliability of the quality assessment was assessed with Cohen’s kappa statistics. Analyses were conducted with the Statistical Package for Social Sciences (SPSS) 27.0.

## Results

### Article characteristics

During the 7-month period of observation, 4,081 articles containing the word “suicide” or “suicides” were retrieved by the automatic search, of which 213 reported on specific instances of suicidal events (Table1). Of these, 168 (78.9%) reported on adult (18 years of age and older) and 45 (21.1%) on adolescent suicides (12–17 years). No article on children under age 12 was found. The three examined newspapers did not differ in the number of articles or in the adult–adolescent distribution. Of the 213 articles, 54% reported on suicide, 17% on attempted suicide, 16% on suspected suicide, and 13% on murder–suicide/attempted suicide (none of these were in adolescence). A greater proportion of the articles about adolescents was found in the national editions (31.7%) than in local editions of the newspapers (14.5%), while the opposite occurred for adult articles (*p* = 0.003) (Table 1).

**Table 1.** Descriptive characteristics of the suicide articles with comparison between adult and adolescent cases in the three newspapers with the largest national circulation in Italy from July 2022 to February 2023

	All	Adults	Adolescents	*p*
Individual suicide cases, *n* (%)	122 (100)	108 (88.5)	14 (11.5)	
Males, *n* (%)	87 (71.3)	82 (75.9)^c^	5 (37.7)	0.004^a^
Females, *n* (%)	34 (27.8)	25 (23.1)	9 (64.3)	
Articles, *n* (%)	213 (100)	168 (78.9)	45 (21.1)	
Articles/suicide cases	1.7	1.6	3.2	<0.0001^a^
Articles reporting on cases of male suicide, *n* (%)	160 (75.1)	133 (83.1)	27 (16.9)	0.007^a^
Articles reporting on cases of female suicide, *n* (%)	52 (24.4)	34 (65.4)	18 (34.6)	
Articles in the surveyed newspapers, *n* (%)				
*Il Corriere della sera*	71 (33.3)	56 (78.9)	15 (21.1)	0.538^a^
*La Repubblica*	82 (38.5)	62 (75.6)	20 (24.4)	
*La Stampa*	60 (28.2)	50 (83.3)	10 (16.7)	
In local editions, *n* (%)	131 (61.5)	112 (85.5)	19 (14.5)	0.003^a^
In national editions, *n* (%)	82 (38.5)	56 (68.3)	26 (31.7)	
Number of words per article, mean (SD)	493.8 (275.7)	462.1 (267.7)	612.5 (275.6)	0.001^b^
Articles by the number of photos, *n* (%)				
No photo	54 (25.4)	44 (26.2)	10 (22.2)	0.693^a^
1 photo	111 (52.1)	85 (50.6)	26 (57.8)	
≥2 photos	48 (22.5)	39 (23.2)	9 (20.0)	

Abbreviation: SD, standard deviation.

a Chi-square or Fisher’s exact test comparing adolescents versus adults; two-tailed *p.*

b Student’s *t-*test comparing adolescents versus adults; two-tailed *p.*

c Sex was not specified in one case.

The newspaper articles covered a total of 108 adult and 14 adolescent individual cases of suicide cases. The ratio articles/suicide cases was greater for adolescents (3.2) than adults (1.6, *p* < 0.0001). The articles reporting on adolescent suicides contained more words (mean 612.5 ± SD 275.6) than articles reporting on adult suicides (462.1 ± 267.7; *p* < 0.001).

Of all the articles, 75.5% reported on male suicides and 24.4% on female suicides (in one case, sex was not specified), with a greater proportion of females among adolescent articles (40.0%) than among adult articles (20.2%, *p* = 0.018). Of the subgroup of 115 articles reporting on suicide death (i.e., excluding articles reporting on alleged or attempted suicide), 78 were in adults (67 males and 11 females) and 37 in adolescents (26 males and 11 females), with a greater proportion of females among adolescents (29.7%) than adult articles (14.1%, *p* = 0.047).

Fifty-five (25.8%) of the articles did not have an accompanying photo, while most (74.6%) contained at least one photo, up to a maximum of five, which were found in two articles (0.9%). There was no statistically significant difference between adult and adolescent articles in the number of photos.

### Adherence to the WHO guidelines for responsible reporting of suicidality

The presence of potentially harmful and protective features according to the WHO guidelines was evaluated for each article with a subsequent comparison between adolescent and adult articles [30] (Table 2). The most frequent harmful element was the suicide method, which was reported in 174 articles (81.7%), with a detailed description in 114 articles (53.5%). A public site as the location of the suicidal act was identified and described in 125 cases (58.7%). Of the 159 articles with an accompanying photo, 76 provided a picture of the suicidal person (35.7% of all the articles). The presence of the word *suicide* in the title was very common (70.9%) and was reported in 151 articles, while less frequently the title informed of the method (31.0%) and accompanying life events (41.8%). In the text of the articles, negative life events as explanation for the suicidal act were found in 150 articles (70.4%). A monocausal explanation for the suicide was present in 61 articles (28.6%) and details from a suicide note in 30 articles (14.1%). Thirty-two articles (15.0%) reported on the effects of the suicide event on bereaved surviving persons, and 30 (14.1%) reported an interview with the bereaved persons (14.1%). Twenty-six articles (12.2%) were positioned in the first page, and 34 (16.6%) within the first three pages (Table 2).

**Table 2.** Potentially harmful and protective features in suicide-related articles in the three Italian newspapers with the largest national circulation from July 2022 to February 2023

Articles, *n* (%)	All, *N* = 213 (100)	Adults, *n* = 168 (100)	Adolescents, *n* = 45 (100)	*p* ^a^
**A**. **Potentially harmful reporting features**				
*Highly prominent placement*				
In the first page	26 (12.2)	19 (11.3)	7 (15.6)	0.440
In the first three pages	34 (16.0)	25 (14.9)	9 (20.0)	0.405
Detailed report of the suicidal act				
Suicide method is reported	174 (81.7)	145 (86.3)	29 (64.4)	**0.001**
Methods is described in detail	114 (53.5)	94 (56.0)	20 (44.4)	0.169
A public site is identified as the place of the suicidal act	125 (58.7)	114 (67.9)	11 (24.4)	**<0.001**
*Reasons for the suicidal act*				
Negative life events related to suicide are reported	150 (70.4)	110 (65.5)	40 (88.9)	**0.002**
Monocausal explanation for suicidality	61 (28.6)	39 (23.2)	22 (48.9)	**0.001**
Details from a suicide note are reported	30 (14.1)	19 (11.3)	11 (24.4)	**0.024**
*Headlines*				
“Suicide” in the headline	151 (70.9)	116 (69.0)	35 (77.8)	0.252
Suicide method in the headline	66 (31.0)	60 (35.7)	6 (13.3)	**0.004**
Life event(s) in the headline	89 (41.8)	58 (34.5)	31 (68.9)	**<0.001**
*About the suicide victim’s family*				
Effects on family members are reported	32 (15.0)	25 (14.9)	7 (15.6)	0.910
Family members are interviewed	30 (14.1)	22 (13.1)	8 (17.8)	0.423
*Photographs*				
Accompanying photo	159 (74.7)	124 (73.8)	35 (77.8)	0.587
Photo of the suicidal person	76 (35.7)	63 (37.5)	13 (28.9)	0.284
**B.** **Potentially protective reporting features**				
*Attribution of suicidal behavior*				
Link with poor mental health is recognized	32 (15.0)	29 (17.3)	3 (6.7)	0.077
Link with substance abuse is recognized	5 (2.5)	5 (3.0)	0 (0.0)	0.242
*The article dispels myths about suicide*				
Dispelling common suicide myths	14 (6.6)	12 (7.1)	2 (4.4)	0.517
*The article draws on health experts, research, and data to inform the public*				
Expert opinion from mental health professional is reported	14 (6.6)	13 (7.7)	1 (2.2)	0.185
Research findings are reported	3 (1.4)	3 (1.8)	0 (0.0)	0.367
Population-level suicide statistics is reported	24 (11.3)	22 (13.1)	2 (4.4)	0.103
*Raises awareness of prevention services*				
Suicide prevention programs are mentioned	8 (3.8)	6 (3.6)	2 (4.4)	0.784
Contact details for suicide support are provided	0 (0.0)	0 (0.0)	0 (0.0)	

a Chi-square test comparing adolescents with adults; two-tailed *p*; *p* values <0.05 are bolded.

Regarding potentially protective features, 32 articles (5.0%) commented on the link between suicide and mental health disorders and 5 articles (2.3%) mentioned substance abuse as a risk factor. Less than 10% of the articles contained other features considered to be beneficial to the public, such as dispelling common misconceptions or false myths about suicide, comments from mental health professionals, or information about suicide prevention programs or services. Only 3 articles (1.4%) referred to research findings and 24 articles (11.3%) included epidemiological data about suicide (Table 2).

When comparing adolescent with adult articles on adherence to the WHO recommendations, statistically significant differences emerged for a few items (Table 2). In adolescent articles, the suicide method and the location were reported less frequently, while negative life events related to suicide, monocausal explanations for suicidality, and details from a suicide note were reported more frequently than in adult articles. The headlines of adolescent articles were more likely than the adult ones to mention the suicide method used (*p* < 0.004) and the life events associated with the suicide (*p* < 0.001) (Table 2).

## Discussion

This systematic examination of all the articles reporting suicide events in the three main newspapers in Italy during a 7-month period identified several noteworthy differences between adolescent and adult suicide reports. First, adolescent suicides were more represented in the press than expected based on population epidemiological data. In fact, suicides in minors account for less than 2% of all suicides [3] but constituted 11.5% of the suicide cases reported in the examined press (p < 0.01). Second, the sex distribution of the adolescent suicides does not reflect the known 1:3 female:male ratio from epidemiological data [3, 43], but there was a greater number of females than males (Table 1), suggesting that female suicides in adolescence are more likely to be reported. This sex difference was not seen in adults where the observed 1:3 female:male ratio is consistent with the epidemiology of suicide in Italy and other countries [1]. Female suicide accounted for 20.4% of the adult articles but for 40.0% of the adolescent ones (*p* = 0.018). Third, each adolescent suicide event had more extensive press coverage than was the case for adult suicides, as indicated by a greater number of articles per case and of words per article (Table 1). The data also show that while most of the suicide articles reporting on adult suicides are published in the local editions of the newspapers, adolescent suicides are more likely to be reported in the national editions (Table 1). This finding further documents the greater visibility of adolescent suicide in the press.

The rarity of death in adolescence, and especially by suicide, may explain why such an occurrence would be considered more newsworthy than in adulthood. More difficult is to explain why female suicide are reported more often than male ones. It is possible that adolescent suicidal behavior be more visible since most suicide attempts are indeed by females. Because, to our knowledge, no similar studies comparing adolescent with adult suicides in the press have been reported, these findings will require replication before generalization to other countries and sociocultural contexts.

The qualitative assessment of the article content revealed heterogeneous adherence to the WHO reporting guidelines [30], with some significant differences between the articles covering adolescent and adult suicides (Table 2). On one hand, the large majority of the articles, both on adolescents and adults, avoided a highly prominent placement in the first three pages of the newspaper. But, on other hand, most articles had one or more elements that are considered potentially harmful, such as a detailed description of the suicidal act, with specifications about the method used, a postulated link between negative life events and suicide, inclusion of the word *suicide* in the headline, and presence of one or more accompanying pictures.

Compared with adults, adolescent articles less frequently reported on the suicide method in the headline displayed the word *suicide* in the headline and reported on the suicide methods or negative life events in the headline, but the article text more often attributed the act to negative life events, offered monocausal explanations rather than considering the complexity of the risk factors for suicide, and included details from a suicidal note (Table 2). With respect to potentially protective reporting practices, only few (0–15%) of the articles included any such features, with no significant difference between adult and adolescent articles. Only 15% of the articles linked suicide to poor mental health, 11.3% provided a perspective from population-level statistics, 3.8% mentioned prevention, and none included contact information for suicide helplines (Table 2).

This relatively low adherence to the guidelines for responsible reporting is consistent with other studies, which, however, did not focus on adolescents [34–37, 44, 45]. For example, in an evaluation of 243 media articles reporting on two high profile cases of suicide, nearly all the articles (99%) breached at least one guideline recommendation and more than half breached three or more recommendations [34]. Better adherence was found in analyses of Australian and Canadian media, with most articles omitting the word *suicide* in the headlines and the suicide method in the text [18, 46].

Particularly problematic seems to be that press articles often lacked information on where to seek help in case of suicide ideation. We found that no article provided contact details for suicide prevention resources and that only 3.8% mentioned suicide prevention (Table 2). The omission seems to be a lost opportunity for prevention as there are suggestions that such information can result in more help-seeking [28]. These findings are consistent with a systematic review that documented that most newspapers over the world do not provide information on where to seek help when covering suicide, with the most favorable rate found in Canada (27%), Ireland (24%), and the Netherlands (17%) [47]. An analysis of U.S. newspapers found poor adherence to guidelines, with most articles providing detailed description of the suicide method and location, but seldom including information about warning signs and risk factors (1% of stories), the role of depression (4%), the role of alcohol (2%), or prevention resources (6%) [37].

From this study, as well as from previous reports, it appears that educational components are seldom included in press articles covering suicide events, thus reflecting the media’s difficulty to take the opportunity to debunk myths and contribute to the effort of suicide prevention. There are indications that using the media to foster suicide prevention can be an effective strategy [9, 29, 48]. Including references to crisis lines or publishing narrative of recovery stories or insert educational topic as relationship between suicide and mental health is indicated [30]. Recent data from a controlled clinical trial suggest that even a brief video presentation about managing suicidality can decrease suicidal ideation and increase help-seeking behavior in adolescents [49]. In a randomized trial conducted in Australia, watching a documentary about the relationship between masculinity issues and mental health was found to increase help-seeking behavior among men [50]. Bringing a song about copying with life difficulties and suicidality to the attention of the general public was found to be associated with a large increase in calls to the US national suicide prevention lifeline, followed by a reduction in suicides [51].

Despite clear indications from the WHO and the other institutions such as the International Association for Suicide Prevention, there is still much to be done in terms of involving media in suicide prevention. Media professionals often have to find a balance between space, deadlines, and the need to sell the product, responding to a big audience that is generally interested in distressing and sensational news. Implementation of responsible reporting by avoiding sensational features can contrast with the urge to get the attention of the public through eye-catching headlines and stimulating details. The desired goal of a progressive and more responsible media reporting on suicide could be reached by working in parallel on increasing public awareness of the risk and protective factors for suicide, as a way of reducing the appeal of negative ways of reporting. The public should eventually become more aware of the negative effects of sensationalizing suicide and more attentive to information about the relationship between suicide and mental health and ways of accessing care. To this end, a strong collaboration between media professionals and health-care professionals is needed. In Italy, a recent collaboration has been started between the Child and Adolescent Neuropsychiatry and the Master Course in Journalism to educate journalist in training in responsible reporting of suicide-related news [52], by providing easily accessible information (on a website) and by doing education of both in training and professional journalists. This seems to be a particularly promising initiative given that the activities are designed and coordinated by a scientific committee composed of both media professionals and child and adolescent’s psychiatrists.

### Limitations

Online media were not included in this study, and this represents a limitation when considering that adolescents tend to access online news more than traditional press media. Differences in adherence to the reporting guidelines were indeed found between online and print media, generally favoring the latter [45]. A second limitation is the 7-month time-frame of the study, which however can be considered sufficiently representative of press reporting. Another limitation is the number of newspapers that were surveyed. However, the three newspapers have the widest distribution nationwide and together account for most of the press circulation in Italy, with a cumulative circulation of more than half a million copies a day. Moreover, no significant heterogeneity was found between these newspapers with respect to the number of articles and age distribution (Table 1). Finally, this was a one-country-only analysis, and the results may not necessarily reflect the situation in other contexts.

## Conclusions

These data suggest that adolescent suicides receive more attention from the press than adult suicides, as shown by a proportionally greater representation than epidemiologically expected, a greater number of articles per suicidal event, and more words per article. Female adolescent suicide, in particular, may be more newsworthy. This study documents generally limited adherence to the WHO media reporting guidelines, suggesting that there is room for improvement in responsible reporting of both adolescent and adult suicide. The special attention of the media to adolescent suicide can become an opportunity for education and prevention. Attempts should be made to understand the perspectives of journalists in reporting suicide and increase the positive contribution that media can give to suicide prevention. To this end, a closer collaboration between adolescent psychiatrists and journalists may be beneficial.

## Supporting information

Davico et al. supplementary material Davico et al. supplementary material

## Supplementary material

The supplementary material for this article can be found at https://doi.org/10.1192/j.eurpsy.2024.2.
