# Supplementary material for: Suicide in the press: an analysis of newspaper coverage of adolescent versus adult suicides in Italy
Source: Eur Psychiatry. 2024 Jan 17;67(1):e9. doi: 10.1192/j.eurpsy.2024.2 (PMC10964272; doi:10.1192/j.eurpsy.2024.2)
Supplement: Davico et al. supplementary material [file S0924933824000026sup001.docx]

| **Supplementary Table 1. Variables collected for each article.** |
| --- |
| From each article the following data were recorded:   - date of publication; - name, sex and age of the suicidal person who committed; - location where the event occurred; - newspaper’s name; - edition (national or local); - type of event (suicide, alleged suicide, attempted suicide, or murder-suicide); - page number on which the news item is reported; - number of words in the article; - number of pictures associated with the article. |
| Categories of potentially harmful factors and definition (according to the WHO recommendations [25]):   - “Visible placement”: stating whether the article was placed on the first or the first three pages; - “Details about the suicidal attempt or gesture”: by examining whether the method was reported and whether there is a detailed account, that is, there are at least two specific details about the act performed; - “Reasons for the gesture”: determining whether the article reported any occurrence that significantly affected the person's life before to the suicide or any occurrence that served as the trigger; whether a monocausal explanation for the gesture was given; and whether the subject's thoughts, whether verbatim or paraphrased, were connected to the act; - “Title”: whether the word "suicide" or any of its synonyms, suicide techniques, or related life events, such poor physical or mental health, are present in the title; - “Family members”: whether the article included interviews with the suicide victim’s survivors, or reported about the impact that the suicide had on them; - “Pictures”: whether the article was accompanied by photos or whether any of the photos feature the person who made the suicide attempt. |
| The following items were used to assess presence of protective factors (according to WHO recommendations[25]):   - “Causes”: whether the link between psychiatric disorders or substance abuse and the suicidal behavior was acknowledged in the article; - “Dispelling Myths”: whether the article attempted to disprove theories that suicide cannot be prevented or mentioned warning signs of a person at risk of committing such an act; - “Experts and data”: whether the article cited the findings of relevant research and epidemiological data or the opinions of mental health specialists like psychiatrists or psychologists. - “Prevention”: whether suicide prevention programs at the local and/or national level were recommended, as well as whether contact information for support services was provided. |
